# Prospectus of the North Carolina Journal of Medicine and Surgery

**Published:** 1857-03

**Authors:** 


### PROSPECTUS OF THE NORTH CAROLINA JOURNAL OF MEDICINE AND SURGERY.

We have received the prospectus of a Journal, bearing the above title, from the Secretary of the North Carolina Medical Society, S. S. Satchwell, M. D., of Long Creek, New Hanover Co., North Carolina.

The new Journal is to be published under the auspices of the Society, and is proposed as an "adjunct in the advancement of Medical Science, and the improvement of the Medical Profession. To this end, it will seek to be a fit medium of expression to the regular physicians of this and other States, who may have written medical communications to make, interesting cases in practice to record, or facts to state of present value or for future reference."

As stated in the prospectus, "Among the thirty-one States of the Union, North Carolina stands almost alone, in having neither a Medical Journal nor Medical College," and we see no good reason why she should not have both; there is, doubtless, the ability in every particular, to sustain such enterprises, if the profession are only united in the purpose to do so. Our friend, the Secretary of the Society, proposing to establish the Journal, would be fully competent, we are sure, to conduct one of high order; and should it be the pleasure of the Society to select him for the post, we should welcome him to the fraternity with much pleasure.

The Secretary is authorised to receive subscribers' names, and we commend the movement, not only to the profession of North Carolina, but the whole Southern country—beyond Virginia or Maryland, at farthest, Southern Journals cannot look for patronage; and from the force of uncontrolable circumstances, compelled to rely upon our own *section* for support, the appeal should not be in vain.

